# Supplementary material for: A Secondary Retrospective Analysis of the Predictive Value of Neutrophil-Reactive Intensity (NEUT-RI) in Septic and Non-Septic Patients in Intensive Care
Source: Diagnostics (Basel). 2024 Apr 16;14(8):821. doi: 10.3390/diagnostics14080821 (PMC11049356; doi:10.3390/diagnostics14080821)
Supplement: Supplementary file 1 [file diagnostics-14-00821-s001.zip › diagnostics-2941584-supplementary.pdf]

Supplementary material

Table S1. Comparison of inflammatory parameters between patients with "renal failure" and patients with "normal renal function."

	**Renal Failure** (n=70)	**Normal renal function** (n=130)	P
**Age**	75.50 [67.00 - 80.00]	71.00 [56.00 - 77.00]	0.006
**Male%**	42.9	54.6	0.150
**Creatinine**	2.12 [1.59 – 3.73]	0.89 [0.69 – 1.25]	< 0.001
**CRP**	21.20 [12.31 - 134.65]	11.37 [2.25 - 32.53]	< 0.001
**PCT**	11.91 [1.02 - 63.71]	0.68 [0.32 - 3.24]	< 0.001
**NEUT-RI**	52.15 [47.95 - 56.48]	48.70 [46.60 - 52.58]	0.002

Table S2: NEUT-RI, PCT and CRP values for the detection of septic and non-septic patients.

	**AUROC (95% CI)**	**Cut-off**	**Sens (95% CI)**	**Spec (95% CI)**	**PPV (95% CI)**	**NPV (95% CI)**
**NEUT-RI**	0.79 [0.73 - 0.86]	50.75	70.9 [60.4 - 79.7]	80.7 [71.6-88.1]	75.6 [65.1-84.2]	77.2 [68.4-84.5]
**CRP**	0.73 [0.66 - 0.80]	7.38	84.6 [75.8-91.2	56.2 [45.5-64.0]	65.8 [56.5-74.3]	77.8 [65.5-87.3]
**PCT**	0.76 [0.69 - 0.84]	2.17	62.9 [52.8 - 74.2]	82.9 [76.4-90]	82.3 [71.2-90]	63.7 [52.9-73.6]

There were not statistically significant differences between AUROC of the three parameters (NEUT-RI vs PCT p=0.83, NEUT-RI vs CRP p=0.29, DeLong's test for two correlated ROC curves). The CRP specificity is statistically different from the NEUT.RI specificity (p=0.01, specificity test for two correlated ROC curves) and from PCT specificity (p=0.007, specificity test for two correlated ROC curves). AUROC= Area Under the ROC curve; Sens = Sensibility; Spec = Specificity; PPV= Positive Predictive Value; NPV= Negative Predictive Value; NEUT-RI= Neutrophil-Reactive Intensity; PCT= procalcitonin; CRP= C-reactive protein

Table S3. NEUT-RI and PCT values for discriminating between "survivors" and "deceased" at 28 days.

	AUROC (95% CI)	Cut-off	Sens (95% CI)	Spec (95% CI)	PPV (95% CI)	NPV (95% CI)
**NEUT-RI**	0.70[0.56-0.83]	53.6	62.5 [39.5-81.3]	76.9 [68.7-82.3]	28.30 [16.8-42.34]	93.0 [87.17-96.76]
**PCT**	0.76 [0.65-0.88]	12.18	76.2 [54.6-90.5]	79.9 [71.5-84.6]	38.09 [23.6-54.3]	95.10 [88.93-98.39]

There were not statistically significant differences between AUROC of the two parameters (p=0.57, DeLong's test for two correlated ROC curves). AUROC= Area Under the ROC curve; Sens = Sensibility; Spec = Specificity; PPV= Positive Predictive Value; NPV= Negative Predictive Value; NEUT-RI= Neutrophil-Reactive Intensity; PCT= procalcitonin

Table S4. NEUT-RI and PCT at ICU admission time, after 48 and 96 hours in septic patients.

	Admission time	48 hours	96 hours	p-value (ANOVA)	P values pairwise comparisons		
					**0h vs 48h**	**0h vs 96h**	**48h vs 96h**
**NEUT-RI (FI) (n=59)**	52.70 [49.65-56.80]	53 [49.00-57.60]	50.50 [47.60-52.90]	0.0002	1	0.0011	<0.0001
**PCT (ng/ml) (n=63)**	6.36 [0.52-36.95]	5.51 [0.90-34.82]	1.65 [0.53-5.74]	<0.0001	0.01	<0.0001	<0.0001

Figure S1. NEUT-RI and CRP depending on the site of infection in "septic" group.

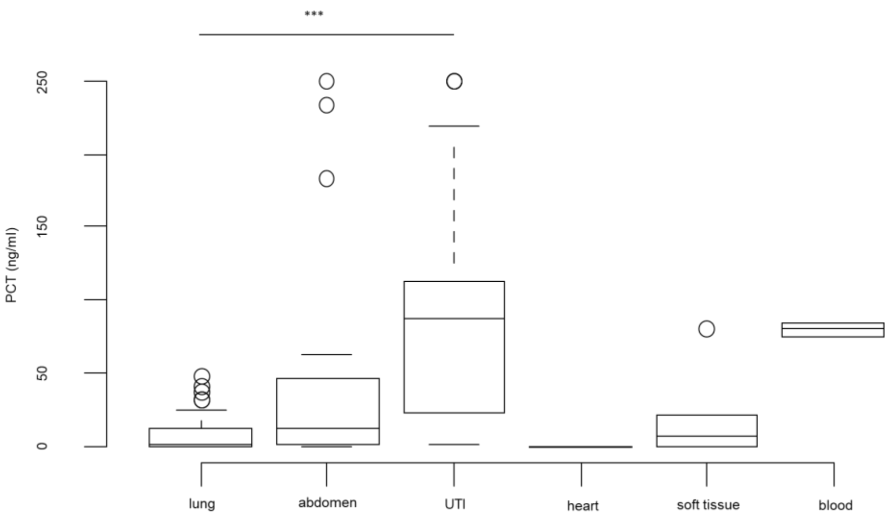

There were no differences in NEUT-RI and CRP depending on the site of infection in "septic" group. There was a statistically significant difference in PCT depending on site of infection ($p<0.001$, Kruskal-Wallis rank sum test), in particular PCT was higher in patients with kidney or urinary tract infection compared to lung infection (88.50 [24.20-113.00] vs 1.64[0.39-12.54], $p<0.001$ Pairwise comparisons using Wilcoxon rank sum test with continuity correction.
